# Numerical Simulation of Segregation in Slabs under Different Secondary Cooling Electromagnetic Stirring Modes

**DOI:** 10.3390/ma17112721

**Published:** 2024-06-03

**Authors:** Daiwei Liu, Guifang Zhang, Jianhua Zeng, Xin Xie

**Affiliations:** 1 Faculty of Metallurgical and Energy Engineering, Kunming University of Science and Technology, Kunming 650093, China; liudaiwei158@163.com; 2 School of Electrical and Information Engineering, Panzhihua University, Panzhihua 617000, China; 3 Pangang Group Research Institute Co., Ltd., Panzhihua 617000, China; zengjianhua68@163.com (J.Z.); xiexin@alu.cqu.edu.cn (X.X.)

**Keywords:** slabs, secondary cooling electromagnetic stirring, alternating stirring, continuous stirring, segregation, numerical simulation

## Abstract

Secondary cooling electromagnetic stirring (S-EMS) significantly impacts the internal quality of continuous casting slabs. In order to investigate the effects of S-EMS modes on segregation in slabs, a three-dimensional numerical model of the full-scale flow field, solidification, and mass transfer was established. A comparative analysis was conducted between continuous electromagnetic stirring and alternate stirring modes regarding their impacts on steel flow, solidification, and carbon segregation. The results indicated that adopting the alternate stirring mode was more advantageous for achieving uniform flow fields and reducing the disparity in solidification endpoints, thus mitigating carbon segregation. Specifically, the central carbon segregation index under continuous stirring at 320 A was 1.236, with an average of 1.247, while under alternate stirring, the central carbon segregation index decreased to 1.222 with an average of 1.227.

## 1. Introduction

The development of steel slab varieties has raised the bar for internal quality standards, particularly emphasizing the homogenization of elemental composition and the refinement of the solidification microstructure [1,2]. However, challenges persist in the production process, such as inadequate isometric crystallinity rates in cast billets and severe segregation of alloying elements, which lead to a diminished steel toughness and plasticity, as well as inconsistent mechanical properties. Therefore, it has become imperative to address elemental segregation in continuous casting production to enhance slab homogenization, ultimately facilitating the production of high-quality steel.

Electromagnetic stirring in the secondary cooling zone during continuous casting (CC) is achieved by installing electromagnetic stirring devices within the secondary cooling zone of a continuous casting machine. The interaction between the stirring magnetic field and the induced electric currents generates Lorentz forces, which compel the flow of liquid metal within the cast slab and facilitate favorable kinetic and thermodynamic conditions conducive to equiaxed crystal growth and solute homogenization. Based on analyses of the carbon content in cast slabs and steel plate properties, Wang et al. [3] optimized the electromagnetic stirring mode and current parameters in the secondary cooling zone, leading to a reduction in the C-type segregation rate in CC slabs. Similarly, Liu et al. [4], through chemical analysis and low-magnification microstructure examination, investigated the effects of electromagnetic stirring frequency parameters in the secondary cooling zone on central segregation and the equiaxed grain rate in CC slabs. Their findings indicated a noticeable alleviation of central segregation in slabs with the adoption of electromagnetic stirring in the secondary cooling zone compared to those without. Dong et al. [5] explored the impact of roller electromagnetic stirring in the secondary cooling zone on the internal quality of Baosteel Q345B, Q550D, and Q345E steel slabs. Industrial experiments revealed significant improvements in the slab’s internal quality post implementation of roller electromagnetic stirring in the secondary cooling zone, with the central equiaxed grain rate increasing from an average of 9.7% to 30.3% and the central C-segregation index decreasing from 1.17 to 1.01. Xie et al. [6] investigated the influence of roller electromagnetic stirring in the secondary cooling zone on the internal quality and rolling properties of high-strength steel. Their findings demonstrated that roller electromagnetic stirring improved central segregation from continuous or semi-continuous A- and B-type segregation to punctate C-type segregation, substantially reducing central segregation or central band-like structures in rolled materials.

Through industrial experiments, Li et al. [7] investigated the impact of electromagnetic stirring in the secondary cooling section of continuous casting on the solidification behavior of high-magnetic-induction silicon steel cast slabs. It was observed that with an increase in the S-EMS current intensity, the center-line segregation of carbon and silicon in the molten steel significantly increased. Yao et al. [8] conducted experiments to study the solidification behavior and composition distribution of 22MnB5 high-strength steel slabs under the influence of S-EMS. The intensity of S-EMS currents gradually increased to 100 A, 150 A, and 300 A, leading to a proportional enlargement of the equiaxed crystal zone. At 150 A, the central carbon segregation in the cast slab was weakest. Jiang et al. [9] investigated the transport phenomena of S-EMS during the continuous casting process of slabs. They established a three-dimensional numerical model and validated its effectiveness by measuring the surface temperature of the cast slab and the intensity of magnetic induction. Ma et al. [10] studied the effect of S-EMS on central segregation in slabs through low-magnification grading of the slab and grading of the hot-rolled coil band structure. It was found that while S-EMS could significantly improve central segregation and central looseness in cast slabs and reduce C-type central segregation, it also led to the presence of noticeable white bands. However, the improvement in band structure was not significant.

However, there is currently a lack of research on the impact of electromagnetic stirring modes on the quality of cast slabs. Mei et al. [11], through numerical simulations, demonstrated that compared to continuous stirring, adopting alternating stirring not only induces periodic variations in the flow within the mold but also significantly reduces the level of liquid surface activity in the mold. In industrial experiments conducted by Chun Liu et al. [12], it was found that continuous stirring resulted in a higher equiaxed grain ratio for 65Mn compared to alternating stirring. Li et al. [13] utilized a three-phase solidification model to numerically predict flow patterns and equiaxed grain ratios during the continuous casting process of three-dimensional full-size thin slabs. They analyzed the impact of alternating electromagnetic stirring reversal periods in the secondary cooling zone on the central equiaxed grain ratio of thin slabs. It was observed that with alternating electromagnetic stirring, the secondary cooling zone exhibited a four-vortex or even five-vortex flow pattern. Additionally, as the reversal period increased, the equiaxed grain ratio initially increased and then decreased. However, these studies have yet to address the influence of electromagnetic stirring modes in the secondary cooling zone on segregation.

The continuous casting process of slab in a specific steel plant is investigated in this study. A comprehensive solidification 3D numerical model is established to investigate the coupled phenomena of flow, heat transfer, and segregation. This study aims to compare and analyze the solute transport behavior induced by different electromagnetic stirring modes and elucidate the influence of electromagnetic stirring on central segregation in slab products.

## 2. Establishment of a Multi-Field Coupling Model

**Figure 1:**
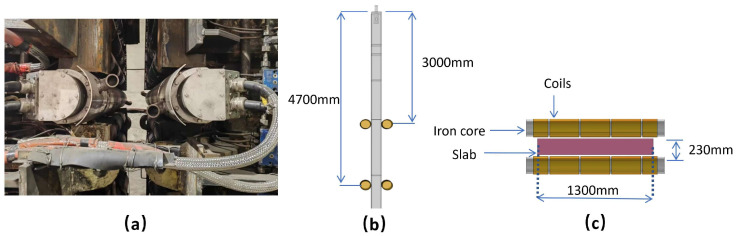
Diagram of the electromagnetic stirring modeling process: (**a**) stirrer field installation; (**b**) roll installation location; (**c**) schematic of a pair of stirring roller models.

The installation positions and a schematic diagram of electromagnetic stirring in the secondary cooling zone of slab casting are illustrated in Figure 1. Based on actual production data from a specific steel plant, a simplified numerical model was established for grade E355 steel. The average composition of this steel grade was obtained through experimental analysis using ICP (inductively coupled plasma) and a carbon-sulfur analyzer, as shown in Table 1. The actual slab casting speed at the factory ranges from 1.1 m/min to 1.3 m/min, and the superheat ranges from 20 K to 30 K. The model values are set at 1.2 m/min and 25 K, respectively. The first pair of electromagnetic stirring rollers is installed at a distance of 3 m from the meniscus, while the second pair is installed at a distance of 4.7 m. Since the stirrers are positioned far from the submerged entry nozzle, the influence of the magnetic field at the nozzle is not considered. The cross-section of the cast slab is depicted in Figure 1c as 230 mm (thickness) × 1300 mm (width). The diameter of the electromagnetic stirring core is 240 mm, with each electromagnetic stirring roller comprising five coils. The inner three coils have 64 turns each, while the outermost two coils on each side have 32 turns.

### 2.1. Model Assumptions

To reasonably simplify the coupling model of flow, heat transfer, solidification, and mass transfer of molten steel during the entire solidification process, the following assumptions are made:

(1) The influence of the molten steel flow on the electromagnetic field is neglected [14], as the magnetic Reynolds number of S-EMS is significantly less than 1 and the electromagnetic field of S-EMS is assumed to be quasi-static due to its low frequency.

(2) The model disregards the Joule heat generated by induced currents, as its magnitude is negligible compared to the latent heat released during steel solidification [15].

(3) The molten steel is treated as an incompressible Newtonian fluid, with constant thermal properties assumed to enhance convergence. Continuous stirring is modeled using time-averaged Lorentz forces rather than transient values.

(4) Turbulent effects in the molten steel are simulated using a low Reynolds number k-ε model, while the mushy zone is treated as a porous medium where the flow satisfies Darcy’s law.

(5) The taper and vibration of the mold are neglected [16].

(6) The effects of steel solidification shrinkage and curvature of the caster on molten steel flow and temperature distribution are disregarded [16].

(7) Considering the significant impact of carbon segregation, the model only accounts for the segregation of carbon.

### 2.2. Governing Equations

To simulate macrosegregation in the slab, it is essential to consider fluid flow, heat transfer, and mass transfer. The main governing equations used in this model are briefly introduced as follows.

(1) Maxwell’s equations [17]:

Ampère’s law:

(1) $$𝛻\times \overset{⃑}{H}=\overset{⃑}{J}+\frac{𝜕\vec{D}}{𝜕t}\approx \overset{⃑}{J}$$

Faraday’s law:

(2) $$𝛻\times \overset{⃑}{E}=-\frac{𝜕\overset{⃑}{B}}{𝜕t}$$

Gauss’s law for magnetism:

(3) $$𝛻\cdot \overset{⃑}{B}=0$$

Ohm’s law:

(4) $$\overset{⃑}{J}=\sigma \overset{⃑}{E}$$

The expression for the time-averaged Lorentz force [18]:

(5) $${\overset{⃑}{F}}_{e}=\frac{1}{2}Re\left(\overset{⃑}{J}\times \overset{⃑}{B^{*}}\right)$$

where $\overset{⃑}{H}$ represents the magnetic field intensity in A/m, $\overset{⃑}{J}$ denotes the current density in A/m^2^, $\overset{⃑}{E}$ represents the electric field intensity in V/m, $\overset{⃑}{B}$ represents the magnetic induction in T, σ is the conductivity in S/m, ${\overset{⃑}{F}}_{e}$ represents the Lorentz force in N/m^3^, Re denotes the real part of a complex number, and $\overset{⃑}{B^{*}}$ denotes the complex conjugate of $\overset{⃑}{B}$.

(2) Continuity equation:

(6) $$𝛻\cdot \left(\rho u\right)=0$$

where u represents the velocity of molten steel in m/s and ρ represents the density of molten steel in kg/m^3^.

(3) Momentum equation:

(7) $$\begin{array}{c} \frac{𝜕}{𝜕t}\left(\rho u\right) \end{array}+𝛻\cdot \left(\rho u_{i}u_{j}\right)=-𝛻P+𝛻\cdot \left\{\mu_{eff}\left[𝛻u_{i}+{\left(𝛻u_{j}\right)}^{T}\right]\right\}+\rho g+F_{r}+{\overset{⃑}{F}}_{e}+S_{p}$$

where ${\overset{⃑}{F}}_{e}$ denotes the original term of the Lorentz force in N/m^3^, $\mu_{eff}$ represents the effective viscosity, which is the sum of laminar viscosity $\mu_{l}$ and turbulent viscosity $\mu_{t}$, and $F_{r}$ represents the thermal-solutal buoyancy force.

(8) $$F_{r}=\rho g\beta \left(T-T_{{\;}_{L}}\right)$$

where β represents the thermal expansion coefficient, k^−1^; T denotes the temperature of the molten steel, k; and $S_{p}$ signifies the Darcy source term.

(9) $$S_{p}=\frac{{\left(1-f_{l}\right)}^{2}}{\left(f_{{\;}_{l}}^{3}+0.001\right)}A_{mush}\left(u-u_{{\;}_{s}}\right)$$

where $f_{l}$ denotes the liquid fraction, $A_{mush}$ represents the coefficient of the mushy zone, taken as $2\times 10^{8}$, and $u_{s}$ signifies the casting speed of the continuous casting slab.

(10) $$f_{l}=1-f_{s}=\left\{\begin{array}{ll} 0 & T\leq T_{s}\\ \frac{T-T_{s}}{T_{L}-T_{s}} & T_{s}<T<T_{L}\\ 1 & T\geq T_{L} \end{array}\right.$$

where $T_{L}$ and $T_{s}$ represent the liquidus temperature and solidus temperature, K.

(4) Low Reynolds number k-ε turbulence two-equation model [19]:

(11) $$\nabla \cdot \left(\rho u\varepsilon \right)+\frac{𝜕}{𝜕t}(\rho k)=\nabla \cdot \left[\left(\mu_{l}+\frac{\mu_{t}}{\sigma_{\varepsilon }}\right)\nabla \varepsilon \right]+C_{1}f_{1}G\rho \frac{\varepsilon }{k}-C_{2}f_{2}\rho \frac{\varepsilon^{2}}{k}+\rho E+\frac{{\left(1-f_{l}\right)}^{2}}{\left(f_{l}^{3}+0.001\right)}A_{mush}\varepsilon$$

(12) $$𝛻\cdot \left(\rho uk\right)+\frac{𝜕(\rho \varepsilon )}{𝜕t}=𝛻\cdot \left[\left(\mu_{l}+\frac{\mu_{t}}{\sigma_{k}}\right)𝛻k\right]+G+\rho D-\rho \varepsilon +\frac{{\left(1-f_{l}\right)}^{2}}{\left(f_{l}^{3}+0.001\right)}A_{mush}k$$

(13) $$G=\mu_{t}\frac{𝜕u_{i}}{𝜕x_{j}}(\frac{𝜕u_{j}}{𝜕x_{i}}+\frac{𝜕u_{i}}{𝜕x_{j}})$$

(14) $$f_{2}=1-0.3exp(-Re_{t}^{2})$$

(15) $$E=2\frac{\mu_{l}\mu t}{\rho }(\frac{{𝜕}^{2}u_{i}}{𝜕x_{j}𝜕x_{k}}{)}^{2}$$

(16) $$D=2\mu_{l}(\frac{𝜕(\sqrt{k})}{𝜕x_{j}}{)}^{2}$$

where k represents turbulent kinetic energy in $m^{2}\cdot s^{-2}$, ε represents turbulent dissipation rate in $m^{2}\cdot s^{-3}$, $\mu_{l}$ is laminar viscosity, $\mu_{t}$ is turbulent viscosity, $C_{1}$ is set to 1.45, $f_{1}$ is set to 1.0, and $C_{2}$ is set to 2.0.

(5) Energy equation:

(17) $$\frac{𝜕(\rho H)}{𝜕t}+\nabla \cdot (\rho uH)=\nabla \cdot \left((+\frac{\mu_{t}}{Pr_{t}})\nabla T\right)$$

(18) $$H=h_{ref}+f_{l}L+\int_{T_{ref}}^{T}\;c_{p}dT$$

where $k_{l}$ represents laminar thermal conductivity in $W\cdot m^{-1}\cdot K^{-l}$, $Pr_{t}$ denotes the Prandtl number, which is set to 0.86, H stands for total enthalpy, $h_{ref}$ is the reference enthalpy, L represents the latent heat of solidification, and $c_{p}$ is the specific heat of the steel liquid in $J\cdot kg^{-1}\cdot K^{-1}$.

(6) Solutes transport equation [20]:

(19) $$\frac{𝜕(\rho c_{i})}{𝜕t}+𝛻\cdot \left(\rho uc_{i}\right)=𝛻\cdot \left[\left(\rho D_{l,i}+\frac{\mu_{t}}{Sc_{t}}\right)𝛻c_{i}\right]+S_{d}+S_{c}$$

(20) $$S_{d}=\nabla \cdot \left[\rho f_{s}D_{s,i}\nabla \cdot (c_{s,i}-c_{i})\right]+\nabla \cdot \left[\rho f_{i}D_{l,i}\nabla \cdot \left(c_{l,i}-c_{i}\right)\right]$$

(21) $$S_{c}=\nabla \cdot \left[\rho (u-u_{s})(c_{l,i}-c_{i})\right]$$

(22) $$c_{i}=c_{l,i}f_{l}+c_{s,i}f_{s}$$

(23) $$c_{l,i}=\frac{c_{i}}{1+f_{s}(k_{i}-1)}$$

(24) $$c_{s,i}=\frac{k_{i}c_{i}}{1+f_{s}(k_{i}-1)}$$

where $Sc_{t}$ represents the Schmidt number, which is set to 1.0 [21]; $S_{d}$ denotes the molecular diffusion source term; $S_{c}$ signifies the convective diffusion source term; $D_{l,i}$ stands for the diffusion coefficient of the solute element in the liquid phase; $D_{s,i}$ represents the diffusion coefficient of the solute element in the solid phase; $c_{i}$ denotes the concentration of the solute element; $c_{l,i}$ is the local average concentration of the solute element in the liquid phase; $c_{s,i}$ is the local average concentration of the solute element in the solid phase; and $k_{i}$ denotes the equilibrium distribution coefficient of element between solid and liquid phases.

### 2.3. The Boundary Conditions

(1) The coil is insulated from the core and the copper mold tube from the slab. Each of the five coil windings of each roller is energized with a two-phase alternating current, with a phase difference of 90° between the currents.

(2) The magnetic field lines are assumed to be parallel to the outer surface of the air unit enveloping the stirrer.

(3) The inlet velocity at the SEN is calculated based on mass conservation by the steelmaking facility. Turbulence-related parameters at the inlet are computed using a semi-empirical equation [22]. The outlet of the computational domain is set as a fully developed boundary.

(4) The SEN walls are assumed to be adiabatic, and the free liquid surface is adiabatic with zero shear stress.

(5) All walls are considered to be no-slip walls, with the wall velocity set to the casting speed. Heat transfer in the mold and secondary cooling zone primarily depends on the position of the slab, as extensively described in our previous work [23].

### 2.4. Simulation Strategy and Process Parameters

**Figure 2:**
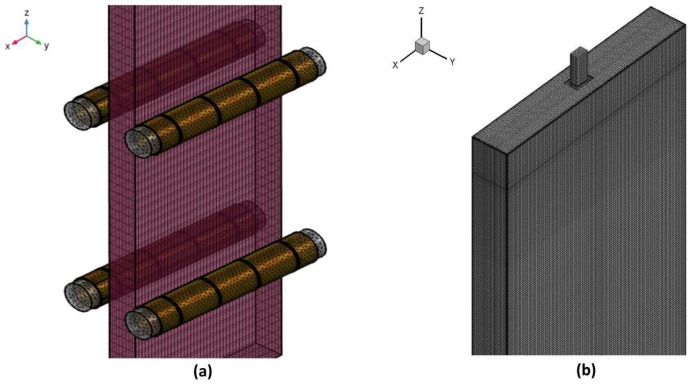
Numerical model mesh diagram: (**a**) mesh of the electromagnetic field model; (**b**) mesh of the full-size model of a slab.

This study divides the computational process into two steps. Firstly, an electromagnetic field numerical model is established using ANSYS APDL, as shown in Figure 2a. Subsequently, a two-phase alternating current is applied to supply power to the five coils of each roller, generating Lorentz forces within the slab. MATLAB interpolation is then used to obtain the Lorentz forces at the fluid calculation grid nodes. Finally, the Lorentz forces are added as momentum source terms to the Navier–Stokes (N-S) equations using a Fluent user-defined function (UDF).

The Fluent computational model, depicted in Figure 2b, includes a 24.8 m long computational domain in the casting direction to ensure complete solidification. Hexahedral structured grids are employed, with grid refinement in the edge regions to obtain accurate solidified shell data. The total number of grids is approximately 3.28 million. The numerical model utilizes the SIMPLE algorithm for iterative calculations, and the momentum equations are discretized using a second-order upwind mode. Convergence criteria are set such that the energy residual is less than 1 × 10^−6^, and other variable residuals are less than 1 × 10^−4^. The material properties used in the calculations are listed in Table 2. Some of the thermophysical properties were determined using JMatPro10.0 (Sente Software Ltd., Surrey, UK), while others were obtained from reference [24].

According to the specific water-cooling method, the corresponding heat transfer coefficient of the cooling zone can be calculated [25] based on the water flow density. The calculated results for each cooling zone are presented in Table 3.

To compare the differences between different stirring modes, simulations and comparative analyses were conducted for two different operating modes of the stirrer, as shown in Table 4. Continuous stirring refers to the continuous application of stirring action, with the current kept stable, ensuring stable Lorentz forces and flow fields. On the other hand, alternate stirring involves the periodic switching of stirring action, resulting in intermittent stirring effects.

By periodically changing the direction of the current supplied to specific coils, it is possible to induce periodic reversal of the Lorentz forces, thereby achieving the transition from the continuous to alternating mode.

## 3. Model Validation

**Figure 3:**
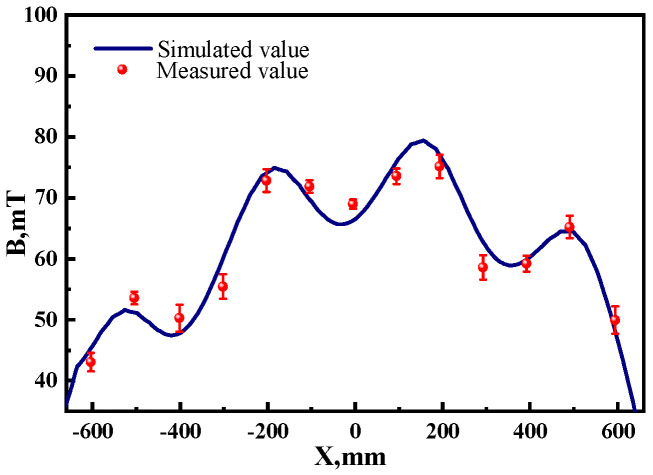
Magnetic induction intensity.

Model validation was achieved through comparison calculation and measurement of magnetic induction. The center-line magnetic induction of the first pair of roller stirrers was measured at the continuous casting site using a KANETECTM-701 Gauss meter (Kanetec Co., Ltd., Nagano, Japan). Figure 3 shows the comparison between the calculated and measured values of magnetic induction along the centerline of the slab cross-section when the current is 320 amperes and the frequency is 5 hertz. The maximum difference between the two values is less than 5%. When the current is 160 A and 240 A at the same frequency, the differences between the calculated and measured values are also below 5%, indicating a good agreement between the measured and calculated values of magnetic induction.

**Figure 4:**
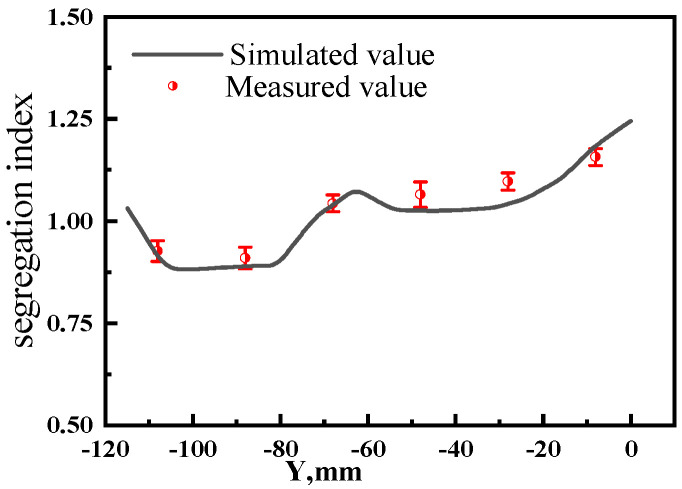
C distribution without S-EMS.

To verify the calculation accuracy of the center segregation model in the slab, carbon segregation was measured without electromagnetic stirring at the center of the slab width. Samples were taken at intervals of 1/2 thickness from the slab center to the surface, with a sampling interval of 20 mm and a drill hole diameter of 5 mm. Six samples were taken for carbon segregation detection. The carbon segregation index (C_i_/C_0_) predicted by the model was compared with the detected carbon segregation index, as shown in Figure 4. The maximum deviation was 4.8%, which is below 5%. The solute carbon segregation predicted by the model is in good agreement with the actual detection results.

## 4. Results and Discussion

### 4.1. Lorentz Force

**Figure 5:**
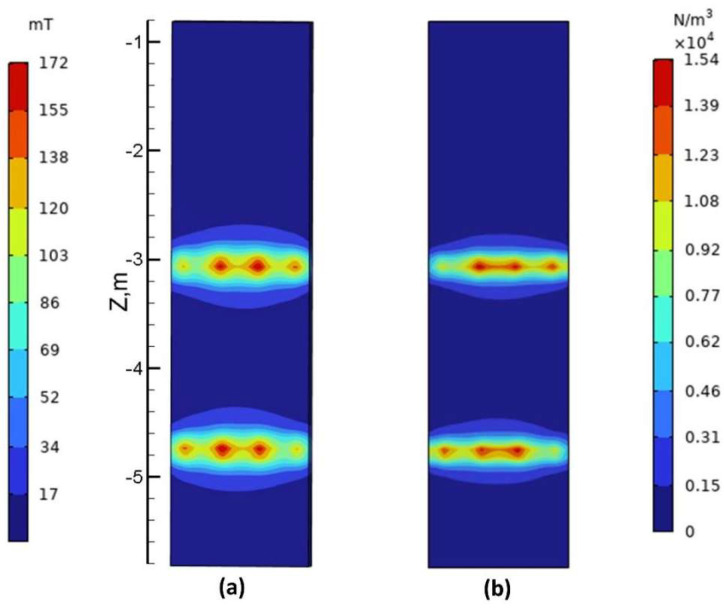
Magnetic induction and Lorentz force distribution on the surface of the slab at a current of 320 A: (**a**) the contour plot of magnetic induction distribution; (**b**) the contour plot of Lorentz force distribution.

Figure 5 illustrates the distribution of magnetic induction and Lorentz force on the surface of the slab at a current intensity of 320 A and a frequency of 5 Hz for Mode A. As depicted in the figure, both the magnetic induction (B) and Lorentz force (F) are concentrated within the secondary cooling zone where the stirrer is located, ranging from Z = −2.5 m to −5.2 m. On the surface of the slab, each pair of stirring rollers exhibits four peaks in both magnetic induction and Lorentz force, with maximum values of approximately 170 mT and 15,000 N/m^3^, respectively. Due to the compact structure of the roller stirrer and its proximity to the slab within the secondary cooling section, where there is minimal gap, magnetic induction is primarily concentrated within a range of 1000 mm around each electromagnetic stirrer.

**Figure 6:**
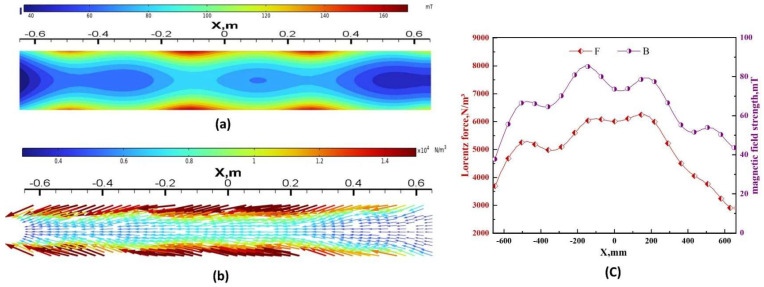
Distribution of B and F in the cross-section (Z = −4.7 m) under continuous stirring with a current of 320 A: (**a**) magnetic induction strength contour plot; (**b**) Lorentz force vector plot; (**c**) numerical values of B and F along the central axis.

The two pairs of rollers on the slab maintain equal but opposite magnetic induction and Lorentz forces from the onset of electrification. Taking the second pair of rollers as an example, the cross-sectional distribution of magnetic induction and Lorentz forces at Z = −4.7 m, with I = 320 A and F = 5 Hz, is depicted in Figure 6. It can be observed that both the magnetic induction (as shown in Figure 6a) and Lorentz force (as shown in Figure 6b) gradually decrease from the surface of the slab to the center. The direction of the Lorentz force is from right to left (pointing towards the negative X-direction), as shown in Figure 6c, and there are four relative peaks of magnetic induction and Lorentz force along the centerline of the slab cross-section. The maximum magnetic field strength is approximately 83 mT, and the maximum Lorentz force is approximately 6200 N/m^3^.

**Figure 7:**
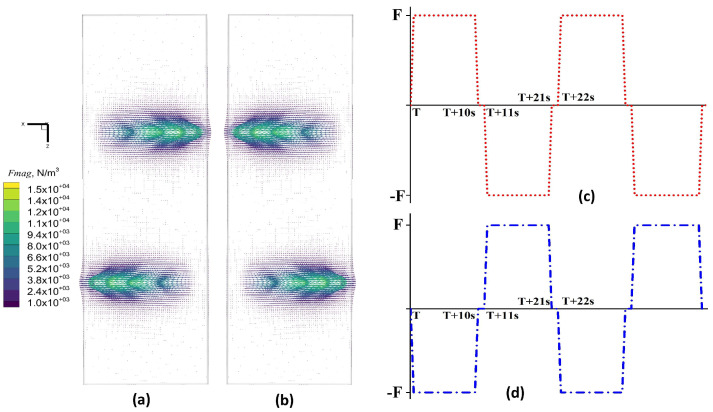
Lorentz force vector distribution and stirring reversal schematics in the central section of the slab at a current of 400 A: (**a**) forward Lorentz force; (**b**) reverse Lorentz force; (**c**) first pair of roll commutation modes; (**d**) second pair of roll commutation modes.

Figure 7 illustrates the vector distribution of Lorentz forces at the center cross-section of the slab and a schematic diagram of stirring reversal for Mode B. The magnitude of the Lorentz force remains constant for both pairs of rolls, while its direction periodically changes. Closer to the stirrer, the Lorentz force exerted on the molten steel is greater. At an electrical current intensity of 400 A and a frequency of 5 Hz, the maximum Lorentz force at the central section is approximately 8000 N/m^3^. Figure 7a,b depict the vector diagrams of the Lorentz force in the forward and reverse directions along the central longitudinal section. After half a cycle, the Lorentz force reverses, decelerating and then accelerating the molten steel in the opposite direction. The roll-type stirrer switches the stirring direction of the molten steel by altering the phase angle of the alternating currents in the two sets of coils. Figure 7c,d illustrate the schematic of the stirring reversal for the first and second pairs of rolls, respectively. In the alternating stirring mode, the reversal cycle (t) is 22 s. The stirring coils generate a forward Lorentz force for 10 s, followed by a 1 s interruption, and then a reverse Lorentz force for 10 s, followed by another 1 s interruption, repeating this cycle.

### 4.2. Flow Field

**Figure 8:**
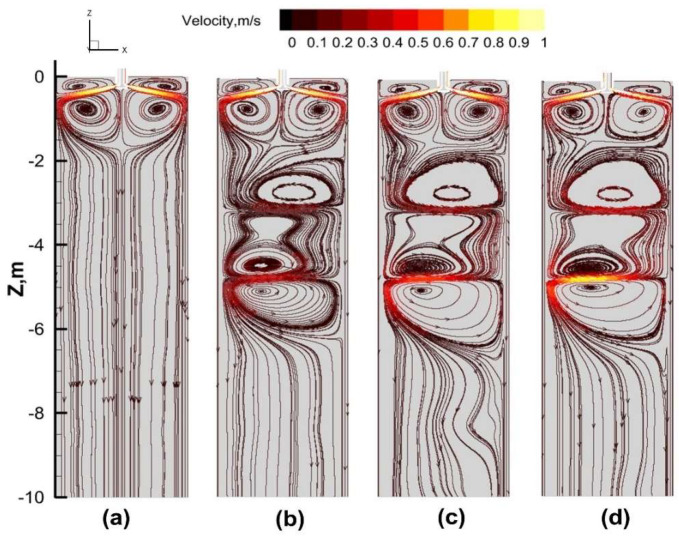
Streamlines and flow field cloud of the longitudinal center section of the slab: (**a**) 0 A; (**b**) 160 A; (**c**) 240 A; (**d**) 320 A.

Figure 8 illustrates the velocity streamlines and flow field contour plots at the center longitudinal section (Y = 0 m) of the slab under different current intensities for Mode A. It is evident that without S-EMS, there is no relative motion between the steel liquid and the solidified shell, resulting in the steel liquid velocity being identical to the casting speed. With the influence of S-EMS, the Lorentz force generated by the electromagnetic stirring roll at −3 m drives the steel liquid to flow from right to left, impacting the narrow face of the mold and ultimately bifurcating into two counter-current flows on either side of the impact point. Due to the Lorentz force induced by the electromagnetic stirring roll at −4.7 m being opposite in direction to that induced by the roll at −3 m, the steel liquid flows from left to right, impacting the narrow face of the mold from the opposite direction. Under the influence of the two pairs of electromagnetic stirring rolls, three vortices are formed, located above the roll installed at −3 m, between the two rolls, and below the roll installed at −4.7 m.

A comparison of Figure 8a–d reveals that with an increasing current intensity, the velocity of the melt at the same position increases accordingly. This is attributed to the increased Lorentz force with a higher current intensity, which enhances the horizontal flow of the steel liquid due to the lateral Lorentz force, thereby altering the original flow path of the downward-moving steel liquid and intensifying the horizontal flow velocity. This simultaneously promotes heat exchange in the stirring zone, facilitating the reduction in overheating.

**Figure 9:**
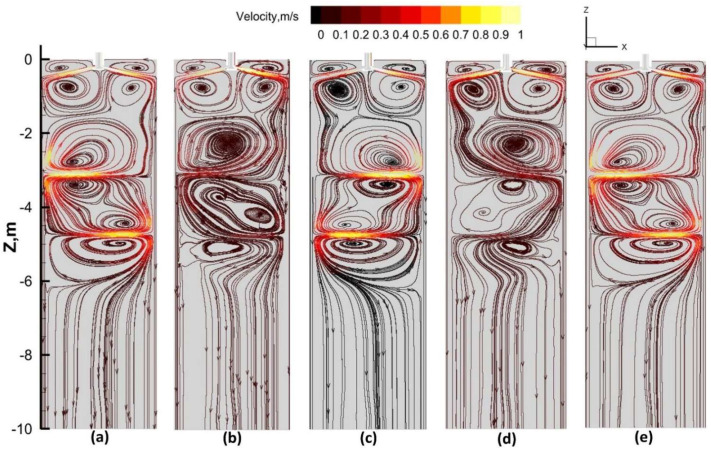
Flow patterns on the cross-section at the slab center during alternating stirring with a current of 320 A: (**a**) t; (**b**) t + 5 s; (**c**) t + 11 s; (**d**) t + 16 s; (**e**) t + 22 s.

Figure 9 illustrates the flow field distribution at a typical moment during one cycle under Mode B with a current intensity of 320 A. From t to t + 5 s, the Lorentz force reverses, exerting a force opposite to the direction of flow, leading to a gradual weakening of the flow intensity. From t + 5 s to t + 11 s, the reverse Lorentz force causes the steel liquid to decelerate to zero before accelerating, resulting in a decrease in flow velocity followed by a reverse increase to maximum velocity. From t + 11 s to t + 16 s, the Lorentz force reverses again, causing a gradual decrease in flow velocity. From t + 16 s to t + 22 s, the reverse Lorentz force leads to a deceleration to zero before accelerating in the opposite direction, with the flow intensity first decreasing and then reversing to accelerate.

In comparison to the continuous stirring mode, the alternating mode results in a continuous and periodic horizontal flow of the steel liquid within the slab. Under the alternating stirring mode, the flow patterns inside the slab exhibit different states, with vortices appearing above and below the stirring rolls.

**Figure 10:**
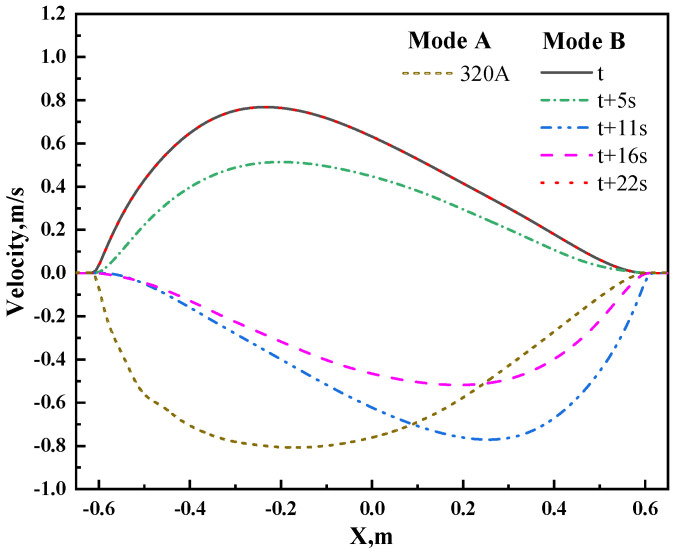
Comparison of transverse velocities at different times for alternating and continuous modes on the centerline of the slab cross-section at Z = −4.7 m with a current of 320 A.

Figure 10 illustrates the velocity distribution along the centerline of the slab cross-section under different stirring modes (current intensity of 320 A, Z = −4.7 m). In the continuous stirring mode, the steel liquid moves in the negative direction of the *X*-axis, reaching a maximum velocity of −0.8 m/s at the position of −0.2 m. In the alternating stirring mode, the velocity exhibits periodic variations over one cycle with five time points. At time t, the steel liquid moves in the positive direction of the *X*-axis, reaching a maximum velocity of 0.8 m/s. From t to t + 5 s, the velocity gradually decreases due to the opposite direction of force relative to the velocity direction, with a maximum velocity of approximately 0.5 m/s. From t + 5 s to t + 11 s, the steel liquid velocity decreases to 0 m/s, then moves in the negative direction of the *X*-axis, reaching a maximum velocity of −0.8 m/s. From t + 11 s to t + 16 s, due to the change in force direction, which opposes the flow direction, the velocity gradually decreases to −0.5 m/s. From t + 16 s to t + 22 s, the steel liquid velocity decreases to 0 m/s, then moves in the positive direction of the *X*-axis, reaching a maximum velocity of 0.8 m/s. Comparing the six curves, it can be observed that the average velocity over time in the alternating stirring mode is lower than that in the continuous stirring mode, indicating a lower flow intensity and consequently a lower stirring effect on the steel liquid.

**Figure 11:**
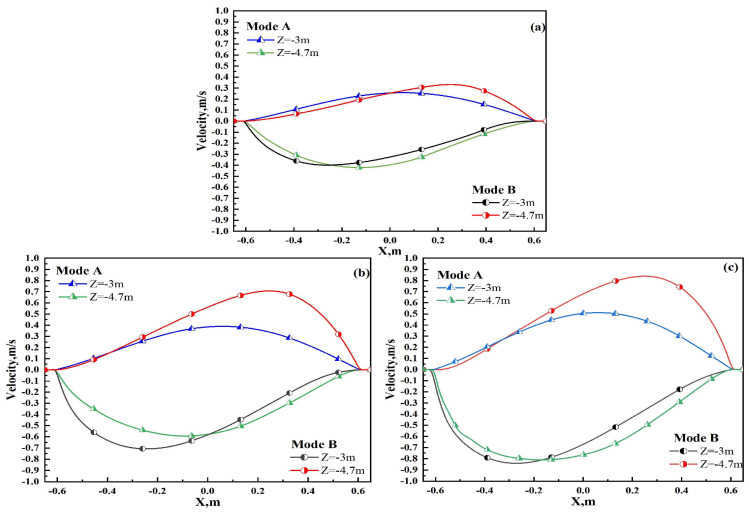
Velocity distribution in the centerline of the cross-section under the roll: (**a**) 160 A; (**b**) 240 A; (**c**) 320 A.

To compare the velocity distributions along the centerline of the slab cross-section under different current intensities (Z = −3 m and Z = −4.7 m) between alternating stirring and continuous stirring, the moment of the maximum velocity in the alternating stirring mode was selected and contrasted with continuous stirring. The results are depicted in Figure 11. For Mode A at Z = −3 m, as the current increases from 160 A to 240 A and then to 320 A, the maximum lateral velocity of the liquid core under the roll decreases from 0.2 m/s to 0.4 m/s and then to 0.5 m/s, respectively. At Z = −4.7 m, the corresponding maximum lateral velocities are −0.4 m/s, −0.55 m/s, and −0.8 m/s as the current increases from 160 A to 240 A and then to 320 A. For Mode B at Z = −3 m, the maximum lateral velocities under the roll decrease from −0.4 m/s to −0.7 m/s and then to −0.8 m/s as the current increases from 160 A to 240 A and then to 320 A, respectively. At Z = −4.7 m, the maximum lateral velocities increase from 0.4 m/s to 0.7 m/s and then to 0.8 m/s after increasing the current from 160 A to 240 A and then to 320 A.

The lateral velocity gradually increases with the current intensity of the stirrer. From Figure 11a, it can be observed that at Z = −3 m, the velocity for Mode B at 160 A is comparable to the maximum velocity at Z = −4.7 m, with a difference of approximately 0.05 m/s, indicating that the horizontal flow velocities of the liquid core under both rolls are symmetrical. Conversely, in continuous mode (Mode A), as shown in Figure 11c, at 320 A and Z = −3 m, the velocity differs significantly from the maximum velocity at Z = −4.7 m, with a difference of approximately 0.3 m/s, indicating asymmetry in the lateral flow velocities of the liquid core under the two rolls. This asymmetry can be attributed to the first roll being closer to the mold, where the flow field is influenced by both the Lorentz force of the stirrer and the mold’s flow field, while the liquid core under the second roll is further away and is not affected by the mold’s flow field. Comparing Figure 11a–c, it can be observed that under Mode B conditions, the lateral flow velocities under both rolls are essentially symmetrical, indicating better uniformity in the flow field.

### 4.3. Carbon Element Segregation

**Figure 12:**
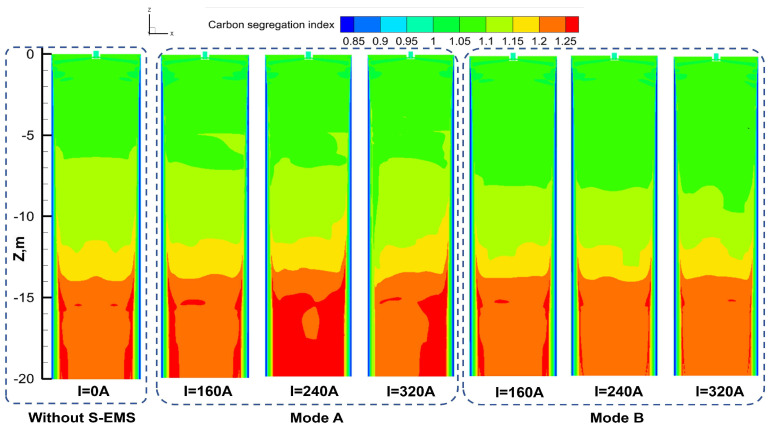
Distribution of carbon concentration at the solidification end of the slab’s center longitudinal section under different S-EMS currents.

Figure 12 illustrates the distribution of carbon segregation in the longitudinal section of the slab center. In the absence of S-EMS, solute diffusion occurs at the solidification end of the slab with a minimal transverse fluid flow, resulting in a relatively uniform solute distribution. Under continuous stirring, i.e., Mode A, the uneven flow promotes carbon enrichment towards the right narrow edge. The significant influence of the three-ring vortex induced by S-EMS, as shown in Figure 8, affects the carbon concentration distribution in the ingot pool. The second pair of electromagnetic stirring rolls generates a greater flow velocity, flushing away carbon elements near the solidification front on the left narrow edge of the ingot and gradually enriching carbon elements on the right narrow edge of the ingot. Concurrently, with the increase in the S-EMS current intensity, the flow velocity of the steel liquid in the ingot increases, resulting in more carbon elements being flushed away from the solidification front and exacerbating the enrichment phenomenon on the right narrow edge. Under alternating stirring, i.e., Mode B, the periodic variation in the flow field leads to an improvement in the unevenness of carbon elements in the width direction. This indicates that alternating stirring can suppress segregation, and with an increase in current, the uniformity of the element distribution improves. At an alternating stirring current of 320 A, the carbon element uniformity is maximized.

**Figure 13:**
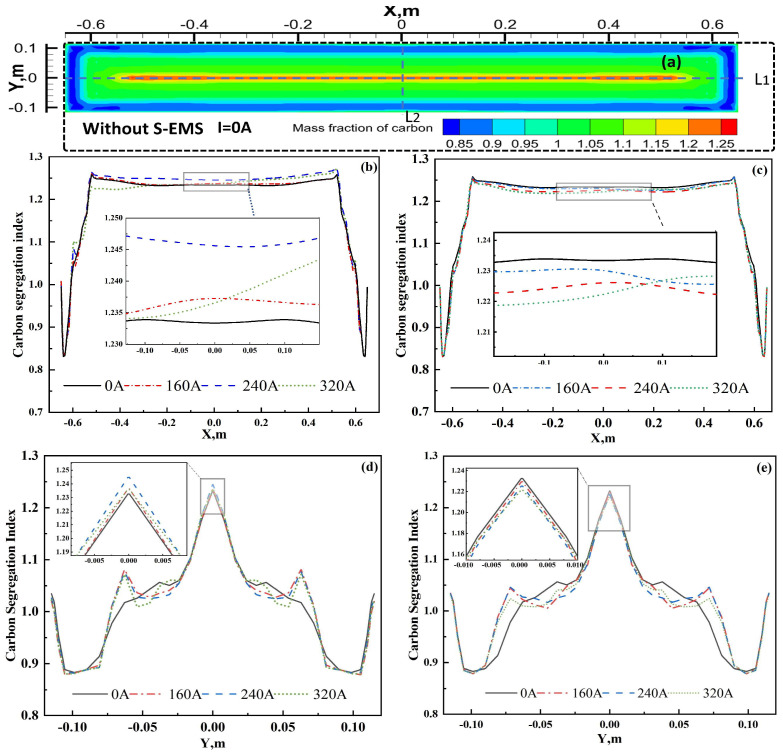
Carbon segregation distribution of centerline in the slab’s cross-section and at different S-EMS currents: (**a**) without S-EMS; (**b**) Mode A of line 1; (**c**) Mode B of line 1; (**d**) Mode A of line 2, (**e**) Mode B of line 2.

The distribution of carbon segregation indices under different S-EMS current intensities is depicted in Figure 13. Figure 13a illustrates the carbon segregation distribution in the transverse section of the ingot without electromagnetic stirring, while Figure 13b,c display the distribution of the carbon segregation index along the thickness direction centerline (L1) for Mode A and Mode B, respectively. Notably, significant negative segregation is observed near the subsurface of the slab due to the rapid steel liquid flow velocity and fast solidification at the mold. Without S-EMS (i.e., I = 0 A), the high degree of solute enrichment in the steel liquid eventually solidifies at the center, resulting in significant positive carbon segregation along the centerline of the ingot, particularly evident at the 1/8 centerline of the wide face where the final solidification occurs (as shown in Figure 12). A comparison between Figure 13b,c reveals a more pronounced transient decrease in carbon element segregation indices at approximately X = −0.57 m and X = 0.57 m for Mode A compared to Mode B. This phenomenon can be attributed to the stronger flow intensity and flushing effect in continuous stirring. Specifically, in the absence of S-EMS, the average carbon segregation index along the centerline (L1) is 1.233, with a difference of 0.026 between the maximum and minimum values. At 160 A, the average carbon segregation index increases to 1.236, and at 320 A, it further rises to 1.247, accompanied by an increased fluctuation in segregation indices. Thus, it is evident that increasing the current exacerbates the average carbon segregation and its fluctuation along the ingot centerline, indicating poorer uniformity compared to the absence of electromagnetic stirring.

Under continuous stirring (Mode A), as the S-EMS current intensity increases, the fluctuation of carbon segregation along the ingot centerline intensifies. The degradation of carbon segregation is most severe near the 1/8 centerline length close to the right narrow face of the ingot due to the imbalance in horizontal flow under the upper and lower pairs of rolls and excessive stirring-induced carbon enrichment at the right narrow edge of the ingot. Consequently, employing continuous stirring (Mode A) with electromagnetic stirring installed in the pre-section of the secondary cooling zone, i.e., the first pair of rolls positioned at Z = −3 m and the second pair at Z = −4.7 m, yields marginal improvements in carbon segregation effects.

Figure 13c demonstrates that under alternating stirring (Mode B), the improvement in carbon segregation is more pronounced with increasing current. The residual of this numerical model is set at 10^−6^. Between X = −0.52 m and X = 0.52 m, the average carbon segregation index decreases to 1.232, 1.228, and 1.227 at 160 A, 240 A, and 320 A, respectively, lower than the value of 1.233 at 0 A. The difference between the maximum and minimum segregation indices at 160 A, 240 A, and 320 A is 0.0302, 0.0299, and 0.0311, respectively, which is closer to the difference at 0 A, indicating a favorable improvement in carbon segregation with an increasing current. However, considering the saturation effect of the magnetic induction intensity of the yoke itself, an excessively high current is not advisable.

The distribution of carbon segregation along the centerline (L2) of the narrow face of the slab under different S-EMS currents is illustrated in Figure 13. A comparison between Figure 13d and 13e reveals that, near the center of the L2 line (Y = 0.00 m), the carbon segregation is highest at 240 A in Mode A, at approximately 1.243, while it is lowest at 320 A in Mode B, at approximately 1.221. Between Y = −0.05 m to −0.075 m and Y = 0.05 m to 0.075 m, the maximum carbon segregation index under Mode B, 1.023, is 0.045 lower than that under Mode A, where it is 1.069. Hence, adopting alternating stirring, i.e., Mode B, compared to continuous stirring, i.e., Mode A, can reduce the average carbon segregation index, facilitating carbon homogenization.

In the aforementioned analysis, the alternating stirring mode in the secondary cooling zone achieves continuous reversal of the stirring direction by periodically changing the direction of the Lorentz force. This enhances the symmetry of the transverse flow field under the stirring rolls, thereby improving the carbon segregation in the slab, with significant enhancement effects observed. This offers a new approach to improving the distribution of carbon elements in the production process by employing alternating stirring in the pre-section of the secondary cooling zone.

## 5. Conclusions

(1) Flow field simulation studies under different stirring modes indicate that in the continuous mode, the lateral velocity of the liquid phase beneath the first and second pairs of rolls is opposite and significantly different. With an increasing current, this difference becomes more pronounced, with a maximum absolute difference in lateral velocity of 0.2 m/s and 0.3 m/s at 160 A and 320 A, respectively. An asymmetry in the internal flow field of the ingot under continuous stirring is observed. In the alternating mode, the maximum lateral velocity of the liquid core beneath the first pair of rolls is approximately equal to that beneath the second pair of rolls, with a maximum absolute difference of approximately 0.05 m/s, indicating a basic symmetry in the lateral velocity of the liquid core beneath the two pairs of rolls.
(2) Simulation studies on the effects of carbon segregation under different stirring modes show that in the continuous mode, the fluctuation in carbon segregation along the centerline of the slab increases with increasing current. Between X = −0.52 m and X = 0.52 m, at 0 A, the average carbon segregation index along the centerline is 1.233, with a difference of 0.026 between the maximum and minimum values, while at 320 A, the average increases to 1.247, with a difference of 0.051 between the maximum and minimum values, indicating an increase in fluctuation. In the alternating mode, the improvement is more significant with an increasing current. Between X = −0.52 m and X = 0.52 m, at 160 A, the average carbon segregation index along the centerline is 1.232, with a difference of 0.0302 between the maximum and minimum values, while at 320 A, the average decreases to 1.227, with a difference of 0.0311 between the maximum and minimum values, indicating minimal fluctuation. Therefore, adopting alternate stirring is advantageous for reducing the average carbon segregation index and promoting carbon homogenization compared to continuous stirring.

## Figures and Tables

**Table 1:** Main chemical composition of E355 steel (wt. %).

C	Si	Mn	P	S
0.17	0.23	0.55	0.013	0.007

**Table 2:** The material properties [24].

Items	Unit	Value
Permeability of vacuum	H·m^−1^	1.257 × 10^−6^
Relative magnetic permeability of steel, coil, and air	/	1
Relative magnetic permeability of iron core	/	1000
Electrical conductivity of steel	S·m^−1^	7.14 × 10⁵
Specific heat capacity of steel	J·kg^−1^·K	680
Thermal conductivity of steel	W·m^−1^·K^−1^	29
Viscosity of steel	kg·m^−1^·s^−1^	0.0055
Density of steel	kg·m^−3^	7020
Solidus temperature	K	1763
Liquidus temperature	K	1802
Latent heat of solidification	J·kg^−1^	270,000
Coefficient of thermal expansion	K^−1^	1 × 10^−4^
Equilibrium partition coefficient of C	/	0.3

**Table 3:** Process parameters of slab continuous casting.

Secondary Cooling Zone	Length (mm)	Heat Transfer Coefficient (Wm^−2^k^−1^)
Mold	800	1200
Zone 1	405	540
Zone 2	555	763
Zone 3	800	629
Zone 4	1730	557
Zone 5	1927	467
Zone 6	3854	400
Zone 7	5806	306
Zone 8	4485	182

**Table 4:** Different electromagnetic stirring modes.

Mode Number	Roller Operating Mode	Frequency	Period	Current (A)
A	Continuous Stirring	f = 5 Hz	/	320,240,160
B	Alternate Stirring	f = 5 Hz	T = 22 s	320,240,160

## Data Availability

The original contributions presented in the study are included in the article, further inquiries can be directed to the corresponding author.
